# Clinical outcomes of combined versus separate carbachol and brimonidine drops in correcting presbyopia

**DOI:** 10.1186/s40662-016-0065-3

**Published:** 2016-12-05

**Authors:** Almamoun Abdelkader, Herbert E. Kaufman

**Affiliations:** 1 Department of Ophthalmology, Faculty of Medicine, Al-Azhar University, Cairo, Egypt; 2 Department of Ophthalmology, Louisiana State University Eye Center, LSU Medical School, New Orleans, LA USA

**Keywords:** Presbyopia, Carbachol, Brimonidine, Depth of focus, Miosis

## Abstract

**Background:**

To test and compare in a masked fashion the efficacy of using a parasympathomimetic drug (3% carbachol) and an alpha-2 agonist (0.2% brimonidine) in both combined and separate forms to create optically beneficial miosis to pharmacologically improve vision in presbyopia.

**Methods:**

A prospective, double-masked, randomized, controlled clinical trial was conducted. Ten naturally emmetropic and presbyopic subjects between 42 and 58 years old with uncorrected distance visual acuity of at least 20/20 in both eyes without additional ocular pathology were eligible for inclusion. All subjects received 3% carbachol and 0.2% brimonidine in both combined and separate forms, 3% carbachol alone and 0.2% brimonidine (control) alone in their non-dominant eye in a crossover manner with one week washout between tests. The subjects’ pupil sizes and both near and distance visual acuities will be evaluated pre- and post-treatment at 1, 2, 4, and 8 h, by a masked examiner at the same room illumination.

**Results:**

Statistically significant improvement in mean near visual acuity (NVA) was achieved in all subjects who received combined 3% carbachol and 0.2% brimonidine in the same formula compared with those who received separate forms or carbachol alone or brimonidine alone (*P* < 0.0001).

**Conclusion:**

Based on the data, the combined solution demonstrated greater efficacy than the other solutions that were tested. Improving the depth of focus by making the pupil small caused statistically significant improvement in near visual acuity, with no change in binocular distance vision.

**Trial registration:**

ACTRN12616001565437. Registered 11 November 2016.

## Background

Presbyopia is the age-related decline in accommodation that diminishes the ability of the eye to focus on near objects [1, 2]. This process usually becomes perceptible between ages 40 and 50 and accelerates with age, necessitating the application of corrective lenses in order to restore near vision [3]. From a pathophysiologic standpoint, multiple theories have been put forth in an attempt to explain this decline in amplitude of accommodation. Changes in the shape, size, and mechanical characteristics of the lens, as well as the function of the ciliary muscle, have all been described [4, 5]. Near visual acuity can be improved by increasing the depth of focus as well as accommodation. A variety of surgical procedures have been considered for restoring accommodation to the presbyopic eye, including surgical expansion of the sclera, using femtosecond lasers to treat the lens or with so-called accommodative intraocular lenses (IOLs) [6]. For accommodation to be restored to the presbyopic eye, it is necessary that the ciliary muscle should still be able to contract with an accommodative effort. There are several lines of evidence that suggest that the ciliary muscle does not atrophy with increasing age and does remain functional; there is no age-related loss of contractility of the isolated rhesus monkey ciliary muscle [7]. Similarly, when the presbyopic eye makes an effort to accommodate, the ciliary muscle contracts [8, 9] despite the fact that the lens shows no accommodative change [9]. Furthermore, the ciliary muscle continues to contract with an accommodative effort even in the pseudophakic eye [10]. Increased depth of focus can come from making the pupil smaller much like a smaller aperture in a camera. The traditional ways for improving vision in presbyopes was through invasive procedures or wearing corrective lenses including pinhole spectacles. Different approaches on the cornea (inlays), the crystalline lens and the sclera are being pursued to achieve surgical correction of this disability [6]. The KAMRA (AcuFocus, Irvine, California, USA) corneal inlay is an annular aperture inlaid in the cornea of one eye where a small entrance pupil is exploited to create a pinhole-type effect that increases the depth of focus and enables improvement in near visual acuity [11–16]. It also reduces the amount of light entering the eye, so the inter-ocular difference in retinal illuminance could alter perceived depth via the Pulfrich effect [17]. Plainis et al. concluded that the Pulfrich effect was not reduced by adaptation, perhaps because the natural pupil diameter of the dominant eye is continually changing throughout the day due to varying illumination and other factors, making adaptation difficult [18]. On the contrary, Ravikumar and Banks [19] concluded that there was some evidence that the KAMRA group experienced a small Pulfrich effect, but the simulated-inlay group experienced a larger and more consistent effect. Thus, KAMRA subjects appeared to have adapted to the reduced illuminance in the treated eye.

We attempted to use drops to approach this effect without surgery. Topical treatment of presbyopia is an attractive approach which, if available and effective, would be the treatment of choice for many patients. Carbachol stimulates the muscarinic and nicotinic receptors on the iris sphincter muscle to create miosis resulting in a smaller pupil aperture that increases the depth of focus. Brimonidine binds to Alpha-2 receptors that are located on the presynaptic nerve endings of the dilator muscle. This binding inhibits further release of the neurotransmitter into the synaptic cleft. This causes reduced activity of the dilator muscle and thereby producing a more miotic pupil. This pilot study aimed to test and compare the efficacy of using a parasympathomimetic drug (3% carbachol) and an alpha agonist (0.2% brimonidine) in both combined and separate forms and individually to create optically beneficial miosis to pharmacologically improve vision in presbyopia.

## Methods

This study was approved by the RCRC Independent Review Board, Austin, Texas, as well as the Ethics Committee of Eye Center and Research, Aseer, Saudi Arabia. Each participant gave written informed consent and the study followed the tenets of the Declaration of Helsinki. The pharmacological stimulation protocol was developed in accordance with that used previously in the invention of Dr. Herbert Kaufman [20].

Participants were randomly selected volunteers. Presbyopia was considered present if an uncorrected end-point print size ≥ Jaeger (J) 5 improved by ≥ 1 optotype with the use of a lens ≥ +1.00 D. All subjects were screened to be in good physical and ocular health and they completed a questionnaire to ascertain any contraindications for participation or predisposition to complications (e.g., heart or respiratory conditions, migraines, high myopia, ocular or systemic medications, or ocular surgeries). All subjects had a fully dilated eye examination before they were considered eligible for the study. The examination screened for contraindications to the drugs, susceptibility to retinal detachment, ocular pathology, or peripheral retinal degeneration. Inclusion criteria were as follows: age between 42 and 58 years, emmetropia [cycloplegic spherical equivalent (SE), ± 0.25 D; astigmatism, ≤ 0.25 D] and binocular uncorrected distance visual acuity ≥ 20/20. Exclusion criteria included patients with myopia, hyperopia and astigmatism higher than 0.25 D as well as those with corneal, lens and vitreous opacities, pupil irregularities, anisocoria, amblyopia, chronic general pathologies and medications that would interact unfavorably with carbachol and brimonidine. None of the patients included in the study had received any topical medication that could cause pupil mydriasis or miosis. During the study, the subjects were closely monitored and regularly asked to report on any ocular, systemic, or physiological reactions they experienced. Atropine was available in the event of adverse effects, although none was reported. All procedures followed were in accordance with the ethical standards of the responsible committee on human experimentation.

### Procedures

A single dose of 3% carbachol together with 0.2% brimonidine in both combined and separate forms and 3% carbachol alone or 0.2% brimonidine alone (control) were instilled in the non-dominant eye of the same ten emmetropic presbyopic subjects with one week washout between tests. In the separate form, carbachol was instilled first followed by brimonidine after 5 min. All of the drugs administered in this study are approved by the US Food and Drug Administration and have been used for years as safe and effective agents for treating ocular pathologies [21–24]. Initial pupil size and both near and distance visual acuities were documented before treatment and at 1, 2, 4, and 8 h after treatment by the same independent examiner in the same room with the same instruments. Distance visual acuity was measured using the standard Snellen projector chart at 4 m. Near visual acuity (NVA) was assessed at 40 cm using a hand-held Rosenbaum chart with Jaeger notation, always employing the same luminosity of 160 cd/m^2^. Pupil size (PS) was measured using Colvard handheld Infrared pupillometer (Oasis Medical, Glendora, CA, USA). Any adverse symptoms and subject satisfaction with near and distance vision were also monitored.

### Statistical analysis

Data analysis was carried out with the Mann-Whitney *U* test, using the MedCalc version 16.8 statistical software. A *P* value of less than 0.05 was considered statistically significant. Data were expressed as mean, range, and standard deviation (SD).

## Results

Ten naturally emmetropic and presbyopic subjects with a mean age of 49.7 ± 4.8 years (range, 42–58 years) were eligible for inclusion. These subjects (6 males and 4 females) with an uncorrected distance visual acuity of at least 20/20 in both eyes were without additional ocular pathology.

In the combined drops group, the mean near visual acuity (NVA) improved significantly from J 8.6 ± 1.5 before treatment to J 1.1 ± 0.3 at 1 h, J 1.1 ± 0.3 at 2 h, J 1.8 ± 0.4 at 4 h and J 2.3 ± 0.5 at 8 h post-treatment (*P* < 0.0001). The mean pupil size (PS) decreased significantly from 4.3 ± 0.5 mm before treatment to 1.2 ± 0.3 mm at 1 h, 1.2 ± 0.3 mm at 2 h, 1.7 ± 0.2 mm at 4 h and 2.1 ± 0.3 mm at 8 h post-treatment (*P* < 0.0001).

In the separate drops group, the mean NVA improved significantly from J 8.6 ± 1.5 before treatment to J 3.4 ± 1 at 1 h (*P* = 0.0002), J 3.6 ± 1 at 2 h (*P* = 0.0002), J 4.5 ± 1 at 4 h (*P* = 0.0004) and J 5.2 ± 0.8 at 8 h (*P* = 0.0008) post-treatment. The mean (PS) decreased significantly from 4.3 ± 0.5 mm before treatment to 1.9 ± 0.3 mm at 1 h, 2.2 ± 0.2 mm at 2 h, 2.5 ± 0.3 mm at 4 h and 2.8 ± 0.2 mm at 8 h post-treatment (*P* < 0.0001).

In the 3% carbachol alone group, the mean NVA improved significantly from J 8.6 ± 1.5 before treatment to J 5.5 ± 1 at 1 h (*P* = 0.001), J 5.9 ± 0.8 at 2 h (*P* = 0.001), J 7 ± 1.2 at 4 h (*P* = 0.007) and J 7.5 ± 1 at 8 h (*P* =0.027). The mean (PS) decreased significantly from 4.3 ± 0.5 mm before treatment to 2.8 ± 0.3 mm at 1 h (*P* = 0.0002), 3 ± 0.3 mm at 2 h (*P* = 0.0002), 3.5 ± 0.3 mm at 4 h (*P* = 0.0007). At 8 h post-treatment, mean (PS) was 4 ± 0.3 mm (*P* = 0.15).

In the 0.2% brimonidine alone group, no statistically significant difference in mean NVA and mean (PS) was found before treatment and at any time point after treatment (*P* > 0.05).

Significant improvement in mean NVA was reported in combined 3% carbachol and brimonidine drops than separate forms or carbachol alone or brimonidine alone (*P* < 0.0001).

**Fig. 1:**
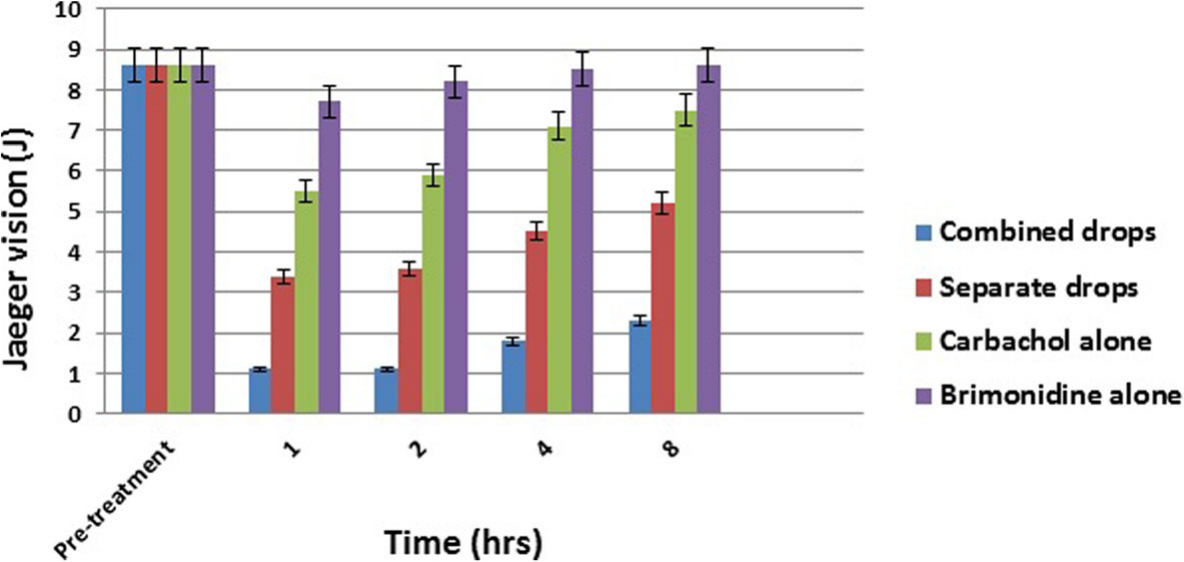
Distribution of mean change in near visual acuity (Jaeger) over time for treatment and control groups

**Fig. 2:**
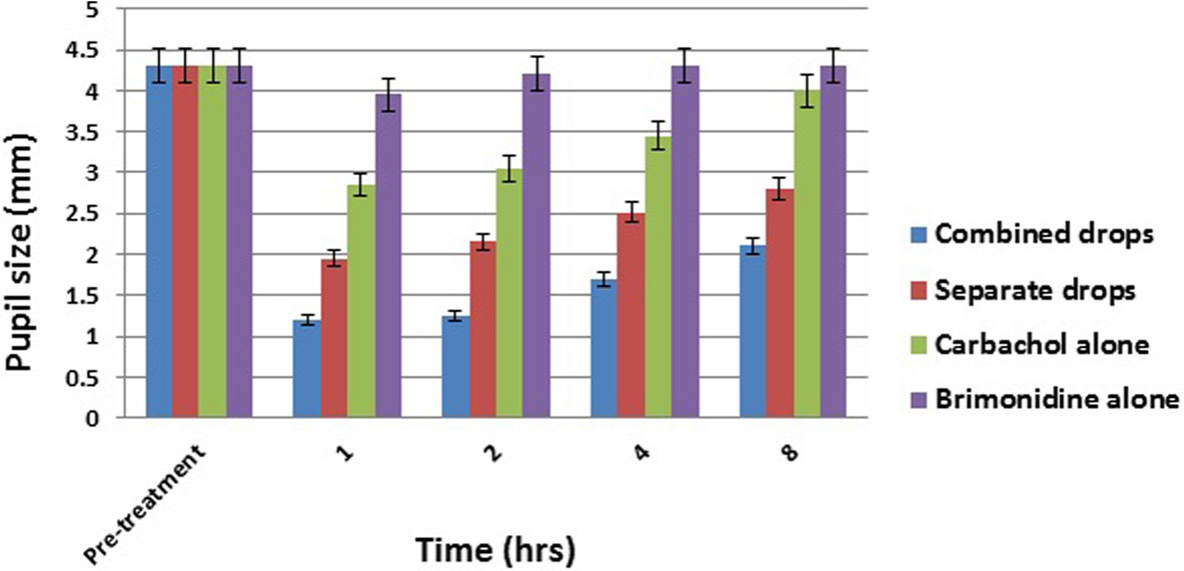
Distribution of mean change in pupil size (mm) over time for treatment and control groups

Data are summarized in Table 1. Figures 1 and 2 show the mean change in near visual acuity (Jaeger) and pupil size (mm) over time for treatment and control groups.

**Table 1:** Mean change in near visual acuity (NVA) (Jaeger) and pupil size (PS) (mm) over time for the same presbyopic subjects receiving combined versus separate 3% carbachol plus 0.2% brimonidine, 0.2% brimonidine alone and 3% carbachol alone

Time		Combined drops	Separate drops	3% Carbachol alone	0.2% Brimonidine alone	*P*-Value*		
						Combined vs. separate	Combined vs. 3% carbachol	Combined vs. 2% brimonidine
Pre-treatment	NVA (J)	8.6 ± 1.5	8.6 ± 1.5	8.6 ± 1.5	8.6 ± 1.5	1	1	1
	PS (mm)	4.3 ± 0.5	4.3 ± 0.5	4.3 ± 0.5	4.3 ± 0.5	1	1	1
1-h	NVA (J)	1.1 ± 0.3	3.4 ± 1	5.5 ± 1	7.7 ± 1.3	*P* < 0.0001	*P* < 0.0001	*P* < 0.0001
	PS (mm)	1.2 ± 0.3	1.9 ± 0.3	2.8 ± 0.3	3.95 ± 0.5	*P* = 0.0006	*P* < 0.0001	*P* < 0.0001
2-h	NVA (J)	1.1 ± 0.3	3.6 ± 1	5.9 ± 0.8	8.2 ± 1.3	*P* < 0.0001	*P* < 0.0001	*P* < 0.0001
	PS (mm)	1.2 ± 0.3	2.2 ± 0.2	3 ± 0.3	4.2 ± 0.4	*P* < 0.0001	*P* < 0.0001	*P* < 0.0001
4-h	NVA (J)	1.8 ± 0.4	4.5 ± 1	7 ± 1.2	8.5 ± 1.4	*P* < 0.0001	*P* < 0.0001	*P* < 0.0001
	PS (mm)	1.7 ± 0.2	2.5 ± 0.3	3.5 ± 0.3	4.3 ± 0.5	*P* < 0.0001	*P* < 0.0001	*P* < 0.0001
8-h	NVA (J)	2.3 ± 0.5	5.2 ± 0.8	7.5 ± 1	8.6 ± 1.5	*P* < 0.0001	*P* < 0.0001	*P* < 0.0001
	PS (mm)	2.1 ± 0.3	2.8 ± 0.2	4 ± 0.3	4.3 ± 0.5	*P* = 0.0006	*P* < 0.0001	*P* < 0.0001

*NVA* = near visual acuity; *PS* = pupil size

*Level of statistical significance: *P* < 0.05

Mild burning sensation was reported in one out of 10 subjects (10%) of the combined drops group compared to 6 subjects (60%) when carbachol drops alone was instilled. Three subjects (30%) in both brimonidine and separate groups reported mild burning sensation. No subject complained of the Pulfrich effect. All subjects in our pilot study reported that they could drive safely day and night without distortions in the perception of any movement.

### Distance visual acuity

The uncorrected distance visual acuity was 20/20 of both eyes in all subjects before treatment and remained at 20/20 at all time periods after treatment in all groups.

## Discussion

This study piloted a simple maneuver aimed at improving near vision in presbyopic subjects. Rather than tackling presbyopia with multifocal or accommodating lenses; pharmacologic treatment relies on the pinhole effect – increasing depth of focus by reducing aperture. The principle is being successfully applied in corneal inlays implanted in the non-dominant eye to enhance near vision. The AcuFocus implant [11] is a corneal implant with a small central artificial pupil. It restores reading vision through increased depth of focus. Although there are some problems with centering the implant i.e., the Pulfrich effect and some surgical complications, it is clear that the principle of a small pupil that moves with the eye can give a good near vision and preserve distance acuity as well [16, 25, 26]. We attempted with drops to approach this effect without surgical interference. In our study, no subject complained of the Pulfrich effect, which occurs due to intraocular differences in retinal illuminance induced by anisocoria. All subjects in our pilot study reported that they could drive safely day and night without distortions in the perception of any movement. However, the Pulfrich effect might have occurred, but that was not objectively tested in this study. In the event that the Pulfrich effect occurs and bothers any subject, they can simply stop taking the drops unlike other surgical procedures that necessitates reversal of the procedure with possible operative and postoperative complications.

In a previous study, a single dose of 2.25% carbachol plus 0.2% brimonidine eye drops was used separately to treat presbyopia and the subjects were followed up for 3 months with no reported ocular complications or tachyphylaxis [27]. The present study used a higher concentration of carbachol (3%) and an alpha agonist (0.2% brimonidine) in both combined and separate forms and individually to improve vision in presbyopia through increasing the depth of focus. Significant improvement in near visual acuity was found to be higher in all subjects who received combined 3% carbachol and brimonidine in the same formula compared with those who received separate forms or carbachol alone or brimonidine alone (*P* < 0.0001). Carbachol, which is formed by substituting a terminal amino group in the acetylcholine molecule, is a quaternary ammonium compound, and so has the pharmacological characteristics of being lipid insoluble, surface inactive, and hydrophilic. Carbachol, therefore, penetrates the cornea very poorly. Corneal penetration by carbachol may be enhanced practically either by reducing the surface tension using benzalkonium chloride as a wetting agent or by administering it in a petrolatum-based ointment and massaging the cornea through closed lids [28]. We attribute this marked improvement in near visual acuity in subjects receiving the combined formula to the penetration enhancers (benzalkonium chloride and carboxymethylcellulose) that were added to the combined formula and perhaps also to the fact that when the receptors of iris dilator and constrictor muscles are both acted upon at the same time, they reinforce each other than when one is stimulated before the other permitting maximal effect with less to overcome. Our results showed that brimonidine tartrate 0.2% alone produced a mild miotic effect mainly during the first hour after instillation under light luminance conditions but this did not reach statistical significance (*P* > 0.05). The effect was similar to that reported in other studies [29, 30]. On the other hand, other studies reported that the antimydriatic effect of brimonidine was pronounced under both light and dark luminance conditions [31–34]. Therefore, the application of brimonidine 20 min before activities in dimly lit areas or at night may be recommended for photic phenomena following laser refractive surgery [35]. In monocular treatment, the vision in the fellow eye with the normal pupil will have some blurry near vision, but distant objects are clear and there is no diminished light perception. When the images are merged, all subjects of treatment group had clear focus at near and distance with no perception of dimness. Carbachol and brimonidine can be used once daily to achieve an 8-h effect.

## Conclusion

The monocular pharmacologic treatment of presbyopia with one drop a day of carbachol and brimonidine in the non-dominant eye permits acceptable reading vision for many presbyopes even in older subjects. This topical agent is noninvasive and, we believe, it meets all of the criteria for an ideal treatment of presbyopia. Based on the data, the combined solution demonstrated greater efficacy than the other solutions that were tested. Despite the small number and the heterogeneity of the patients involved in this pilot study, its findings suggest that this treatment is very promising. Additional studies are planned in the future to use the drops in presbyopia with different refractive errors as in hyperopic and myopic presbyopes and in different situations such as pseudophakic and postlasik presbyopes.
